# Assessing the impact of climate change and a water management programme on white sturgeon physiology in the Nechako River, British Columbia

**DOI:** 10.1093/conphys/coaf014

**Published:** 2025-03-08

**Authors:** Muhammed A Oyinlola, Mostafa Khorsandi, Rachael Penman, Madison L Earhart, Richard Arsenault, Steve McAdam, Colin J Brauner, André St-Hilaire

**Affiliations:** Centre Eau Terre Environnement, Institut National de la Recherche Scientifique, Canadian Rivers Institute, 490 Couronne St, Québec City, QC G1K 9A9, Canada; Institute for the Oceans and Fisheries, University of British Columbia, 2202 Main Mall, Vancouver, BC V6T 1Z4, Canada; Centre Eau Terre Environnement, Institut National de la Recherche Scientifique, Canadian Rivers Institute, 490 Couronne St, Québec City, QC G1K 9A9, Canada; Department of Zoology, University of British Columbia, 6270 University Boulevard, Vancouver, BC V6T 1Z4, Canada; Department of Zoology, University of British Columbia, 6270 University Boulevard, Vancouver, BC V6T 1Z4, Canada; Hydrology, Climate and Climate Change Laboratory, École de technologie supérieure (ÉTS), 1100 Notre-Dame St W, Montréal, QC H3C 1K3, Canada; Applied Freshwater Ecology Research Unit, BC Ministry of Water Land and Resource Stewardship, University of British Columbia, 2202 Main Mall, Vancouver, BC V6T 1Z4, Canada; Department of Zoology, University of British Columbia, 6270 University Boulevard, Vancouver, BC V6T 1Z4, Canada; Centre Eau Terre Environnement, Institut National de la Recherche Scientifique, Canadian Rivers Institute, 490 Couronne St, Québec City, QC G1K 9A9, Canada

**Keywords:** Climate change, conservation, hydrothermal impact, Nechako River, thermal exposure risk, white sturgeon

## Abstract

Climate change is impacting river ecosystems, underlining the need for water management strategies to protect native species within these ecosystems. Here, we evaluate the impact of climate change and water management on the physiology of white sturgeon (*Acipenser transmontanus*) in the Nechako River, British Columbia (Canada). Using the CEQUEAU hydrological–thermal model, we simulated daily water temperatures from 1980 to 2099 under two climate scenarios (SSP2-4.5 and SSP5-8.5). We assessed thermal exposure risk (*T_e_*) for different developmental stages of white sturgeon, focusing on the warmest 6-month period. Our findings show that embryos and yolk-sac larvae exhibit resilience, with *T_e_* values consistently <1 under both scenarios, signifying low thermal stress. In contrast, feeding larvae and juveniles experience elevated *T_e_* values, indicating significant future thermal stress. For feeding larvae, *T_e_* values exceeded 1 under both scenarios, reaching up to 1.5 by the mid-century (2050s) and up to 1.8 by the end of the century (2090s) under SSP5-8.5. Juvenile white sturgeon also faced increased thermal risks, with *T_e_* values rising >1 during July and August, reaching 1.4 and 1.8 by the 2050s and 1.8 and 2.0 by the 2090s under SSP5-8.5, compared to the 1980s. These results underscore the need to evaluate the existing water management programme to better accommodate the projected changes in thermal conditions associated with climate change. Additionally, regulated river discharge, which can both increase and decrease downstream temperatures, offers a strategic opportunity to mitigate some climate impacts through strategic dam discharge management.

## Introduction

Climate change is altering riverine ecosystems, emphasizing the need for water management to ensure the health and survival of endemic aquatic species. River water management plays an important role in regulating water quality, quantity and the overall health of aquatic ecosystems, which are affected by changes in temperature, pH, dissolved oxygen, nutrient levels and pollutant concentrations (Carron and Rajaram, 2001; Richter *et al.,* 2003; Song *et al.,* 2019; Zarri *et al.,* 2019; Bernhardt *et al.,* 2022). Effective water management strategies protect and restore aquatic habitats, such as riparian zones, wetlands, and other ecosystems, ensuring that they continue to support endemic species (Richter *et al.,* 2003). These management strategies often regulate discharge in river systems to balance societal demands with the ecological needs of endemic species. Adapting water management plans to address the evolving impacts of climate change has the potential to enhance the resilience and sustainability of aquatic ecosystems.

Water temperature has profound effects on aquatic animals across levels of biological organization, from chemical reactions and metabolism to species distributions (Guderley, 2004; Clarke and Pörtner, 2010; Halsey *et al.,* 2015; Eliason and Anttila, 2017). Changes in water temperature directly impact an organism’s overall physiological performance, behaviour and survival. Water temperature impacts chemical reactions and reactions kinetics, which are fundamental to physiological processes. Processes such as digestion and respiration are influenced by water temperature up to an optimal level, beyond which their efficiency declines, impacting the rates of these processes and organismal health (Sardella *et al.,* 2004; Schulte, 2015; Muñiz-González and Martínez-Guitarte, 2020). The effects of water temperature on an individual’s physiology can have broader implications for population dynamics and ecosystem interactions. Interactions between water temperature and other environmental factors can either favour or impede organismal reproduction and growth, ultimately shaping the distribution and abundance of species (Perry *et al.,* 2005; Fernandes *et al.,* 2020).

The Nechako white sturgeon (*Acipenser transmontanus*) is endangered due to recruitment failure resulting from the effects of river regulation on critical spawning habitats (DFO, 2014; McAdam *et al.,* 2005). Persistent recruitment failure since 1967 will cause the adult population, currently estimated at 553 individuals, to decline to 200 fish within 30–64 years (van Poorten *et al.*, in review), with hatchery inputs serving as a temporary measure to prevent extirpation. White sturgeon have specific thermal requirements that influence their growth, metabolism and reproductive success (Jay *et al.,* 2020; Penman *et al.,* 2023). Elevated water temperatures, driven by reduced water discharge due to regulation and climate change, can disrupt these physiological processes (Earhart *et al.,* 2023) potentially leading to recruitment failure. Consequently, recruitment challenges may also result from habitat degradation caused by substrate alterations resulting from changes in river discharge (Boucher *et al.,* 2014; McAdam, 2012). Ongoing and future increases in water temperature pose a substantial threat to the recovery and long-term sustainability of white sturgeon populations. Understanding these thermal impacts is essential for developing effective conservation and management strategies to ensure the resilience of white sturgeon in a changing climate.

**Figure 1:**
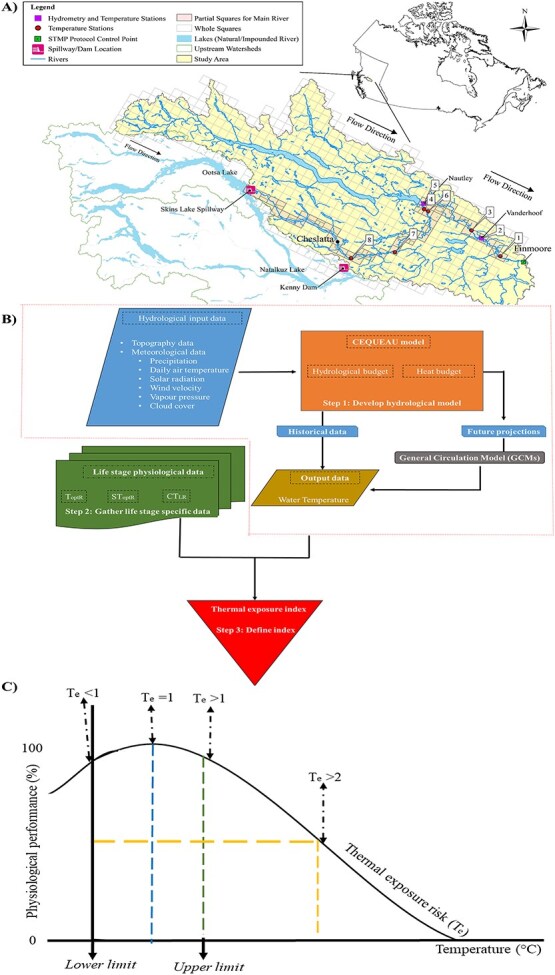
A) Nechako watershed showing the Skins Lake spillway and hydrological stations. B) A schematic diagram of the framework adopted from Oyinlola *et al.* (2023) used in this study. C) Thermal Performance Curve (TPC) showing the relationship between water temperature and fish performance. The blue dashed line (first vertical line) represents the optimal temperature where the performance is at its peak (*Te* = 1). Thermal exposure risk (*Te*), refers to the likelihood of adverse effects due to water temperature extremes, with *Te* values >1 indicating greater risk and worse performance (*Te* > 1). The green dashed line (second vertical line) indicates a moderate *Te* where performance begins to decline. The yellow dashed line (third vertical line) represents higher *Te*, resulting in a significant decrease in performance (*Te* > 2). The performance of the fish declines at temperatures lower and higher than the optimal, reflecting the fish’s reduced efficiency and increased thermal stress.

The Nechako River system in central British Columbia, Canada (Fig. 1A), presents a unique case study for evaluating the effects of climate change and water management on riverine ecosystems. This river is characterized by regulated discharge, significant out-of-basin water diversion, and its location in a northern region, making it particularly susceptible to climate change impacts. Northern regions are experiencing warming at a faster rate than the global average due to Arctic amplification, compounded by extreme weather events that result in prolonged droughts and heavy precipitation (Bush, 2022; Rantanen *et al.,* 2022). The river supports endangered species, including sockeye salmon (*Oncorhynchus nerka*) and white sturgeon, highlighting the importance of targeted water management to ensure species survival. One notable water management plan is the Summer Temperature Management Program (STMP), implemented in the early 1980s to regulate water releases from the Kenney Dam to maintain downstream river temperatures <20°C during the sockeye salmon spawning migration period from 20 July to 20 August (Macdonald *et al.,* 2012). Although this programme has effectively prevented water temperature from exceeding 20°C at the Vanderhoof station (Macdonald *et al.,* 2012), its focus on sockeye salmon highlights the need for modifications to address the conservation of other species that may differ in their thermal sensitivities.

Recent studies (Earhart *et al.,* 2023; Khorsandi *et al.,* 2023; Oyinlola *et al.,* 2023, 2024; Gatien *et al.,* 2024) emphasize the need to ensure that water temperature management programmes in regulated rivers can address the needs of diverse species and life stages, particularly under changing climatic conditions. Continuous monitoring and analysis of these programmes, alongside the assessment of their effectiveness for other migratory and resident species, is essential. This approach ensures the programme continues to accommodate shifting ecological demands and water resource requirements, ultimately contributing to the resilience and sustainability of the river ecosystem including the Nechako River.

In this study, we employed an established framework (Oyinlola *et al.,* 2023) (Fig. 1B) to assess the impact of climate change on the white sturgeon in the Nechako River. We used a hydrological and river temperature model called Centre Québécois des Sciences de l’Eau (CEQUEAU) to simulate daily water temperatures from 1980 to 2099. Using physiological data from the literature for different developmental stages of white sturgeon, we developed a thermal exposure index. We projected the thermal exposure risk for the warmest 6-month period each year, including the water release management period, under two climate change scenarios (SSP2-4.5 and SSP5-8.5) by the middle and end of the century.

## Materials and Methods

We employed a combined modelling framework (Oyinlola *et al.,* 2023) to evaluate the potential impact of climate change on the white sturgeon in the Nechako River. This framework integrates a hydrological model with physiological data specific to each life stage to assess thermal exposure risk (*T_e_*), which represents the likelihood of adverse effects on the physiological performance and survival due to water temperature extremes, with *Te* values >1 indicating greater risk and worse physiological performance (Fig. 1C) (Oyinlola *et al.,* 2023). We hypothesized that the *T_e_* of the white sturgeon would rise above the optimal value of ‘1’ under climate change. First, we used CEQUEAU to simulate the Nechako River’s daily historical water temperature from 1980 to 2019. Next, we developed the thermal exposure index, a quantitative measure of water temperature exposure over time, using white sturgeon physiological data. Last, we projected the *T_e_* for the warmest 6-month period of the year (May–October), which includes the water release management period (20 July–20 August), under two contrasting climate change and socio-economic scenarios: SSP2-4.5 and SSP5-8.5 by mid (the 2050s) and end of the century (the 2090s).

### CEQUEAU model

CEQUEAU is a semi-distributed hydrological–thermal model used for simulation and forecasting flow and water temperature (Morin and Couillard, 1990; Morin and Paquet, 1995) (see supplementary information for details). It employs a two-step discretization to spatialize hydrological processes within the watershed area. Each grid square (called CE) is subdivided into a maximum of four sub-areas (polygons called CPs), which act as a hydrological response unit and for which vertical routing and water storage are conceptually represented through three interconnected reservoirs.

In implementing CEQUEAU, parameter calibration is a critical step. This calibration process involves two steps: first, calibrating the hydrological module using observed streamflow data from hydrometric stations along the Nechako River, and then calibrating the thermal module using water temperature gauges located between the Kenney Dam and Vanderhoof. Manual calibration was initially performed to define the parameter domain, followed by the implementation of an automatic calibration algorithm. The covariance matrix adaptation evolution strategy (CMA-ES) is used for this purpose. CMA-ES has been extensively compared to other optimization algorithms for model calibration in hydrology, consistently demonstrating superior performance in finding global optima and achieving faster convergence (Arsenault *et al.,* 2014).

### Thermal tolerance limits and critical habitat for Nechako white sturgeon

We used data from our previous studies on white sturgeon thermal tolerance during each early life stage (i.e. embryo, yolk-sac larvae, feeding larvae and juvenile) (Earhart *et al.,* 2023; Penman *et al.,* 2023). However, for the feeding larvae stage, physiological data and literature were limited, highlighting an urgent need for further studies to fill this data gap. Hence, we relied on expert opinion to define the thermal limits and evaluated scenarios assuming a threshold similar to either the preceding life stage (14–18°C) or the subsequent life stage (14–20°C). Expert opinion was obtained through consensus among co-authors with relevant expertise in sturgeon physiology.

We defined the Optimal Temperature Range (T_optR_), Sub-optimal Temperature Range (ST_optR_) and Critical Thermal Limit Range (CT_LR_) for each early life stage (embryo, yolk-sac larvae, feeding larvae and juvenile) based on the laboratory findings and expert opinion (Table 1). T_optR_ refers to the temperature range in which the fish’s physiological performance is at its peak, while ST_optR_ represents the range where some critical functions are lost, but <25% mortality is observed. CT_LR_ is the range where >50% mortality occurs. We calculated the *T_e_* using *T_optR_*, *ST_optR_* and *CT_LR_*.


(1) \begin{equation*} {T}_{ei}=0, if\ \left[{T}_a,{T}_b\ \right]<{T}_{optR} \end{equation*}

(2) \begin{equation*} {T}_{ei}=1, if\ \left[{T}_a,{T}_b\ \right]\subset{T}_{optR} \end{equation*}

(3) \begin{equation*} {T}_{ei}=2, if\ \left[{T}_a,{T}_b\ \right]\subset{ST}_{optR} \end{equation*}

(4) \begin{equation*} {T}_{ei}=3, if\ \left[{T}_a,{T}_b\ \right]\subset{CT}_{LR} \end{equation*}

where *T_ei_* is the thermal exposure risk for cell i; T_a_ and T_b_ are the minimum and maximum temperature ranges, respectively.

**Table 1:** White sturgeon thermal exposure risk (*T_e_*) applied for this study, where a value >1 indicates an elevated *T_e_* beyond optimal conditions

**Life stage**	**Variable/Parameter**	**Description**	**Temperature range (°C)**	**Thermal exposure risk (*T***_***e***_ **)**	**Reference**
Embryo		Temperature below optimal temperature (growth/general health condition)	<14	0	(Earhart *et al.,* 2023).
	T_optR_	The optimal temperature (growth/general health condition)	14–18	1	(Earhart *et al.,* 2023).
	ST_optR_	Sub-optimal temperature (loss of some critical function and <25% mortality)	>18	2	(Earhart *et al.,* 2023)
	CT_LR_	Critical temperature (total loss of critical function and >50% mortality)	NA	NA	
Yolk-sac larvae		Temperature below optimal temperature (growth/general health condition)	<14	0	(Earhart *et al.,* 2023)
	T_optR_	The optimal temperature (growth/general health condition)	14–20	1	(Earhart *et al.,* 2023)
	ST_optR_	Sub-optimal temperature (loss of some critical function and <25% mortality)	>20	2	(Earhart *et al.,* 2023)
	CT_LR_	Critical temperature (total loss of critical function and >50% mortality)	NA	NA	
Feeding larvae **(18°C)**		Temperature below optimal temperature (growth/general health condition)	<14	0	Expert opinion
	T_optR_	The optimal temperature (growth/general health condition)	14–18	1	Expert opinion
	ST_optR_	Sub-optimal temperature (loss of some critical function and <25% mortality)	>18	2	Expert opinion
	CT_LR_	Critical temperature (total loss of critical function and >50% mortality)	NA	NA	
Feeding larvae **(20°C)**		Temperature below optimal temperature (growth/general health condition)	<14	0	Expert opinion
	T_optR_	The optimal temperature (growth/general health condition)	14–20	1	Expert opinion
	ST_optR_	Sub-optimal temperature (loss of some critical function and <25% mortality)	>20	2	Expert opinion
	CT_LR_	Critical temperature (total loss of critical function and >50% mortality)	NA	NA	
Juvenile		Temperature below optimal temperature (growth/general health condition)	<14	0	
	T_optR_	The optimal temperature (growth/general health condition)	14–18	1	(Wang *et al.,* 1985, Wang *et al*. 1987, DFO, 2014, Cheung, 2019)
	ST_optR_	Sub-optimal temperature (loss of some critical function and <25% mortality)	>18	2	(Wang *et al.,* 1985, Wang *et al*. 1987, Hildebrand *et al*. 2016)
	CT_LR_	Critical temperature (total loss of critical function and >50% mortality)	NA	NA	

**Figure 2:**
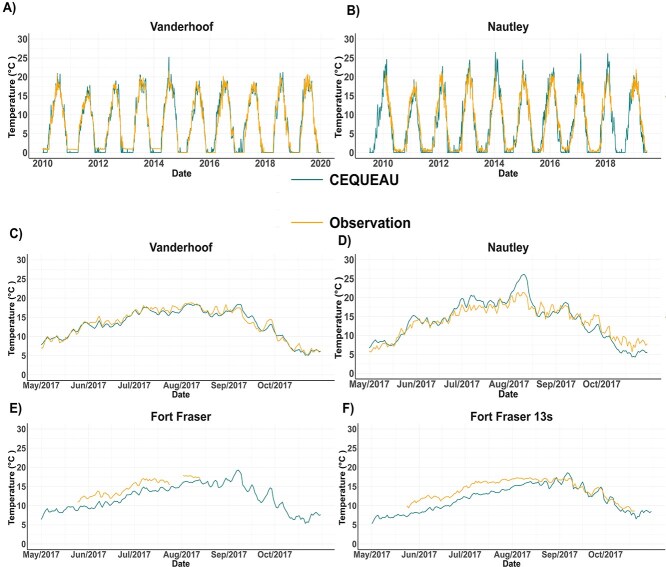
Comparative analysis between the simulated water temperature data from the CEQUEAU model and historical water temperature records sourced from the Canadian Climate Archive (https://climate.weather.gc.ca/historical_data/search_historic_data_e.html) for four stations along the Nechako River. (A) Time series of CEQUEAU-simulated water temperatures and historical observed water temperature data for the Vanderhoof station, 2010–19. (B) Time series of CEQUEAU-predicted water temperatures and historical observed water temperature data for the Nautley station, 2010–19. (C) Focused plot for the Vanderhoof station, May–October 2017. (D) Focused plot for the Nautley station, May–October 2017. (E) Focused plot for the Fort Fraser station, May–October 2017 (gap where historical water temperature data were not available). (F) Focused plot for the Fort Fraser 13 s station, May–October 2017.

### Climate and socio-economic scenarios

We projected the future changes in Nechako River water temperature using data from eight General Circulation Models (GCMs) as part of the Coupled Model Intercomparison Project Phase 6 (CMIP6) (Eyring *et al.*, 2016) (Supplementary Table S1). We examined two sets of combined climate and socio-economic scenarios. The first scenario, SSP2-4.5, represents an intermediate pathway where the world follows historical social, economic and technological trends. The second scenario, SSP5-8.5, describes a high-emission trajectory characterized by rapid economic growth, reliance on competitive markets, innovation and participatory societies (Riahi *et al.,* 2017). By using these GCMs and scenario combinations, we aimed to capture a range of possible future outcomes for the Nechako River water temperature. The incorporation of multiple models and scenarios enabled a comprehensive assessment of uncertainties associated with climate projections.

### Analysis 

We analysed the modelled mean water temperature across the Nechako watershed from 1980 to 2099. We focused on the sections of the river where white sturgeon are distributed. Further, our analyses prioritized the six hottest water temperature months in the Nechako watershed (May–October). We then concentrated on the period when each life stage is present within the Nechako River and analysed the life stage-specific spatio-temporal pattern of *T_e_* for the Nechako River for the embryo, 15 May–24 June; yolk-sac larvae, 24 May–7 July; feeding larvae, 20 June–31 July; and juvenile, 1 May–31 October.

We estimated the average water temperature and *T_e_* in each 0.005 × 0.005°C cell calculated from simulated daily water temperature data. To account for variations among climate models, we determined the average *T_e_* across all GCMs and assigned a numerical value to each *T_e_* category (*T_e_* 0 = 0, *T_e_* 1 = 1, *T_e_* 2 = 2, *T_e_* 3 = 3). We calculated the average *T_e_* for the historical period (1980s, averaging 1980–89) and future periods—mid-century (2050s, averaging 2050–59) and end-century (2090s, averaging 2090–99)—under two emission scenarios: SSP2-4.5 and SSP5-8.5. Similar to the previous study (Oyinlola *et al.,* 2023), we also analysed the average *T_e_* in white sturgeon critical habitats in the Nechako River watershed as identified under Canada’s Species at Risk Act (SARA) (Fisheries and Canada, 2014; SARA, 2002). Finally, we analysed the *T_e_* for white sturgeon’s early life stages (embryo, yolk-sac larvae, feeding larvae and young-of-the-year juveniles) for the period when each life stage is known to exist in the Nechako River. For the feeding larvae stage, we analysed the two variations in optimal temperature ranges denoted here as feeding larvae18 (optimal range of 14–18°C) and feeding larvae20 (optimal range of 14–20°C).

## Results

### CEQUEAU model evaluation

We evaluated our water temperature model results using the Root Mean Square Error (RMSE). RMSE is a metric that quantifies the average magnitude of discrepancies between predicted and actual values. We tested predicted against the actual values from four stations along the Nechako River, Vanderhoof, Nautley, Fort Fraser and Fort Fraser 13 s (Fig. 2). The RMSE values for these stations are as follows: 1.27 (Vanderhoof), 1.68 (Nautley), 2.41 (Fort Fraser) and 2.43°C (Fort Fraser 13 s).

### Climate trends in the Nechako River

**Figure 3:**
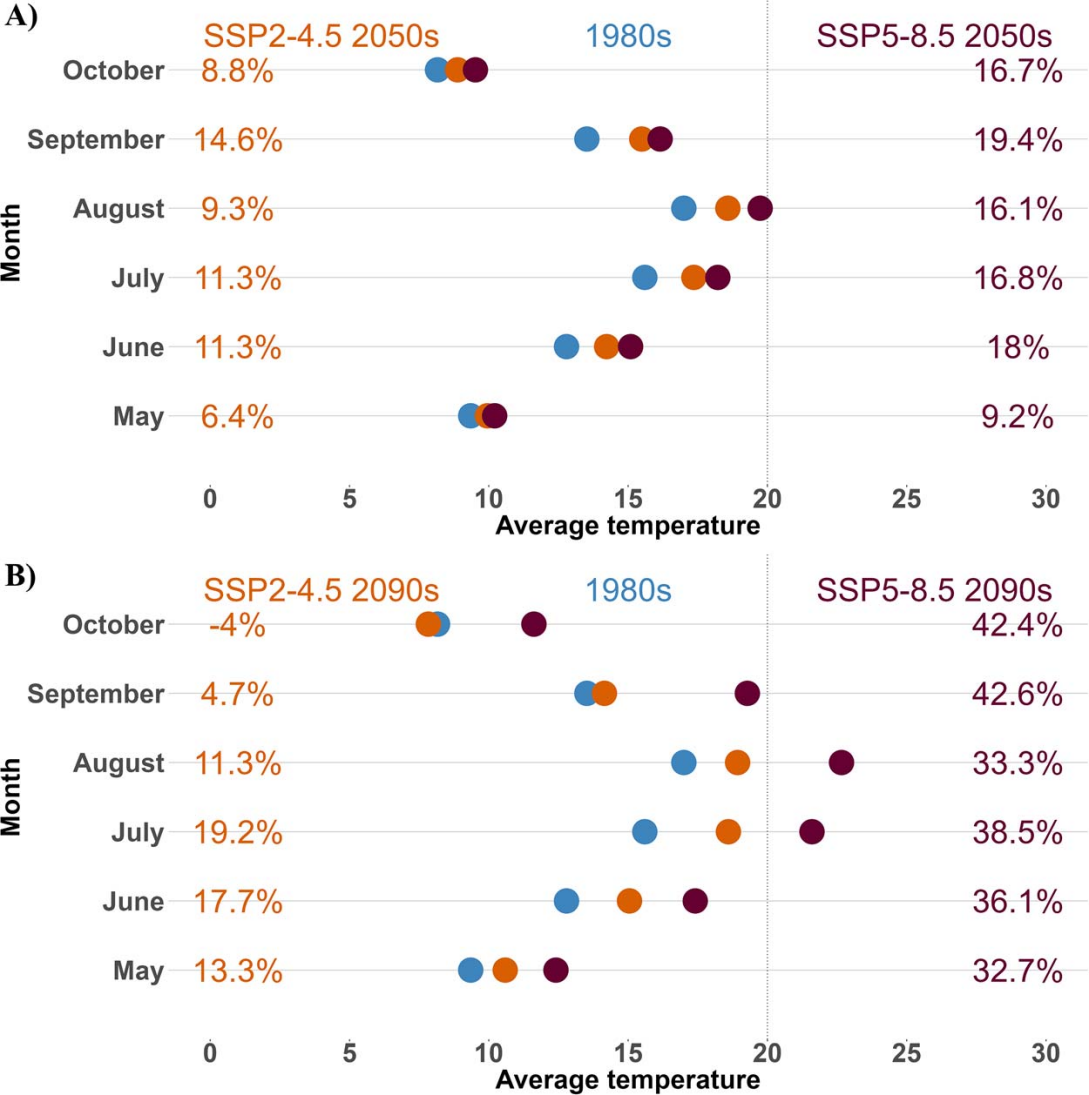
Nechako River temperature during the months examined in this study in the 1980s (average between 1980 and 1989) and under climatic–socioeconomic scenarios; SSP2–4.5 and SSP5–8.5 by A) the mid-century (2050s, average between 2050 and 2059) and B) end-century (average between 2090 and 2099). Percentage change in water temperature relative to the 1980s under both scenarios is indicated on the y-axis as a relative indicator of the water temperature change. The dotted line indicates the recommended water temperature threshold of 20°C.

Our results show that the average water temperature across the Nechako River within the 6-month evaluation period ranged from 8.2 to 17°C in historical years—1980s (average from 1980 to 1989) with the lowest water temperature in October and the highest in August (Fig. 3A and B). However, under climate change scenarios SSP2-4.5, by the mid-century (the 2050s, average from 2050 to 2059), the Nechako River temperature was projected to increase to 18.6°C ± 1.2 (mean ± standard deviation (SD)) in the historical hottest month of August, indicating an increase of 9.3% relative to 1980s, while under SSP5-8.5, we projected an increase of 16% (to 19.7°C ± 1.4). Our results also show that the largest increase in water temperature would occur in September with an increase of 15% (to 15.5°C ± 0.3) and 19% (to 16.2°C ± 0.3) under SSP2-4.5 and SSP5-8.5, respectively (Fig. 3A).

By the end of the century (2090s, average from 2090 to 2099) under the SSP2-4.5 scenarios, our results show that August will remain the hottest month in the Nechako River, with the projected increase in water temperature to 18.9°C ± 3.3, an 11.4% increase relative to the 1980s. However, under SSP5-8.5 in the same time frame, the water temperature is projected to increase to 22.7°C ± 1.5, a 33% increase (Fig. 3B). Furthermore, under SSP2-4.5, September water temperature would increase by a minimal 5% while October water temperature would decrease by 4% relative to the 1980s. However, our results indicate that the Nechako River temperature in September and October would increase considerably by 42%, reaching 11.62 and 19.3°C, respectively, under SSP5-8.5.

### Changes in thermal exposure risk (T_**e**_) of Nechako white sturgeon by the mid and end of the century relative to the 1980s

#### Changes in thermal exposure risk (T_**e**_) across the Nechako River

**Figure 4:**
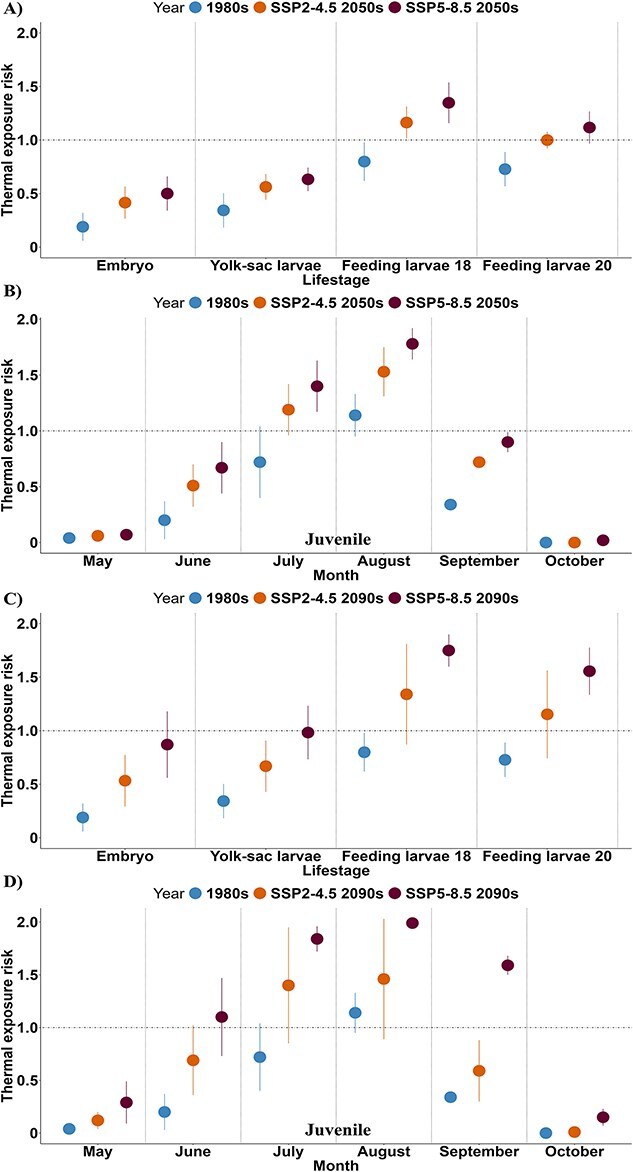
The thermal exposure risk of white sturgeon embryo, yolk-sac larvae, feeding larvae and juvenile life stages for the period when the life stages are present in the Nechako River under SSP2–4.5 and SSP5–8.5. For embryo, yolk-sac larvae and feeding larvae (larvae18 and larvae20) life stages by the A) mid-century (2050, average between 2050 and 2059) B) end of the century (2099s, average between 2090 and 2099) relative to the 1980s (average between 1980 and 1989). For juvenile life stage by the C) mid-century (2050, average between 2050 and 2059) D) end of the century (2099s, average between 2090 and 2099) relative to the 1980s (average between 1980 and 1989) for May–October. Error bars represent SD.

Overall, our findings show that future T_e_ will consistently remain <1 for periods when each embryo and yolk-sac larvae are present in the Nechako River (i.e. 15 May–24 June and 24 May–7 July for embryo and yolk-sac larvae, respectively) under SSP2-4.5 and SSP5-8.5 by the 2050s (Fig. 4A). These results are comparable to historical *T_e_* values estimated for the 1980s. In contrast, both assumed thermal risk ranges for feeding larvae (feeding larvae18 and feeding larvae20), led to *T_e_* values >1 under both scenarios by the 2050s (Fig. 4A). Similar to the 2050s, we projected that the embryo and yolk-sac larvae *T_e_* by the 2090s will be <1 under both scenarios considered in this study (i.e. SSP2-4.5 and SSP5-8.5) (Fig. 4B). However, for the feeding larvae life stage, we projected *T_e_* values >1 under both scenarios similar to historical *T_e_* values estimated for the 1980s (Fig. 4B).

For the juvenile life stage, *T_e_*, was predicted to exceed 1 during July (1.2) and August (1.6) under SSP2-4.5 by the 2050s relative to the 1980s (Fig. 4C). Under SSP5-8.5 in the same time frame, *T_e_* values were projected to be >1 in July (1.4) and August (1.8) compared to the 1980s. However, by the 2090s compared to the 1980s, the juvenile life stage, *T_e_*, was projected to rise >1 in July (1.4) and August (1.5) under SSP2-4.5 (Fig. 4D). Under SSP5-8.5, we projected *T_e_* to increase >1 in June (1.1), July (1.8), August (2.0) and September (1.6) by the 2090s compared to the 1980s.

#### Changes in thermal exposure risk (*T*_*e*_) in critical habitats

**Figure 5:**
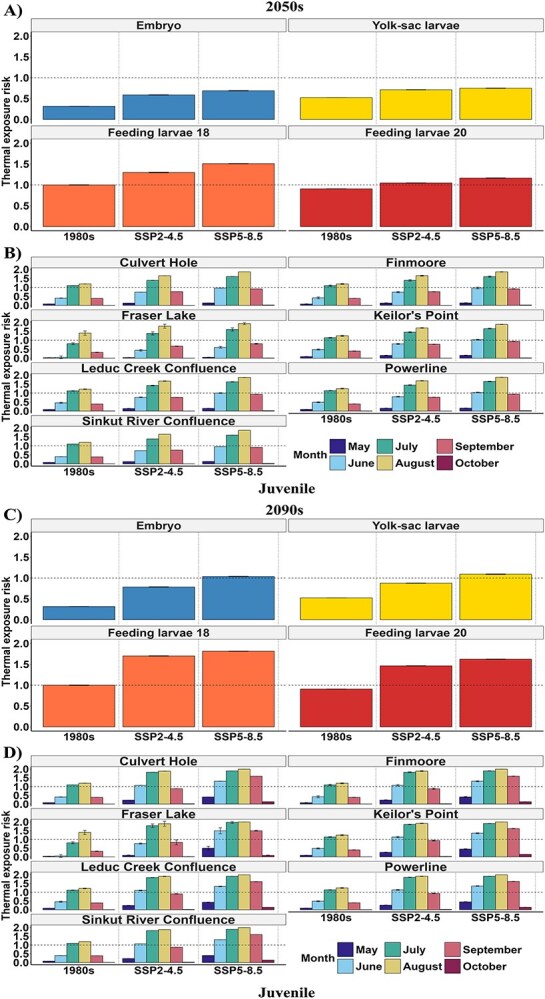
The white sturgeon of the Nechako River early life stages (embryo, yolk-sac larvae, feeding larvae and juvenile) critical habitats thermal exposure risk for the 1980s (average 1980–89) and under climate change scenarios; SSP2–4.5 and SSP5–8.5 in the 2050s (average between 2050 and 2059s) and 2099s (average between 2090 and 2099) from May to October. A and C) For Embryos, Yolk-sac larvae and feeding larvae in Vanderhoof Braided Section for the period when the life stages are present in the Nechako River. B and D) For Juveniles in other habitats from May to October. The long dashed line indicated a thermal exposure risk of 1. Error bars represent SD.

**Figure 6:**
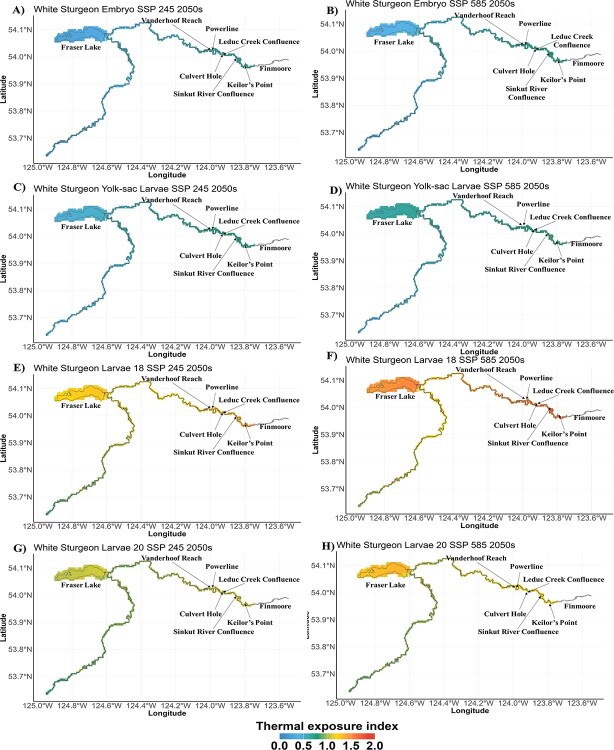
Thermal exposure risk spatial map for white sturgeon of the Nechako River life stages; embryo, yolk-sac larvae and larvae under SSP2–4.5 and SSP5–8.5 in the 2050s (average 2050–59). (A, B) For the embryo life stage occurring between 15 May and 24 June. (C, D) For the yolk-sac larvae life stage occurring between 24 May and 7 July. (E, F, G, H) For the larvae life stage occurring between 20 June and 31 July. Cool and warm colours represent low and high thermal exposure risk, respectively.

Our analysis shows that the projected *T_e_* values would remain <1 by the 2050s under both SSP2-4.5 and SSP5-8.5 for embryos and yolk-sac larvae in the Vanderhoof Reach (Figs 5A and 6A–D). For the feeding larvae life stage, irrespective of the optimal temperature variations considered, the *T_e_* values were projected to exceed 1 in both climatic scenarios, although feeding larvae20 under SSP2-4.5 scenarios would only marginally exceed 1 by the 2050s (Figs 5A and 6E–H). The juvenile life stage utilizes a broader range of habitats than the other life stages (Fig. 1C). Our projections show that by the 2050s compared to the 1980s when the juvenile *T_e_* value was only <1 in Fraser Lake, the *T_e_* levels would remain consistently above a value of 1 in all critical habitats during July and August, irrespective of the scenario (SSP2-4.5 and SSP5-8.5) (Fig. 5B). In addition, under the SSP5-8.5, our results show that *T_e_* would be >1 at Keilor’s Point and Powerline sections in June.

**Figure 7:**
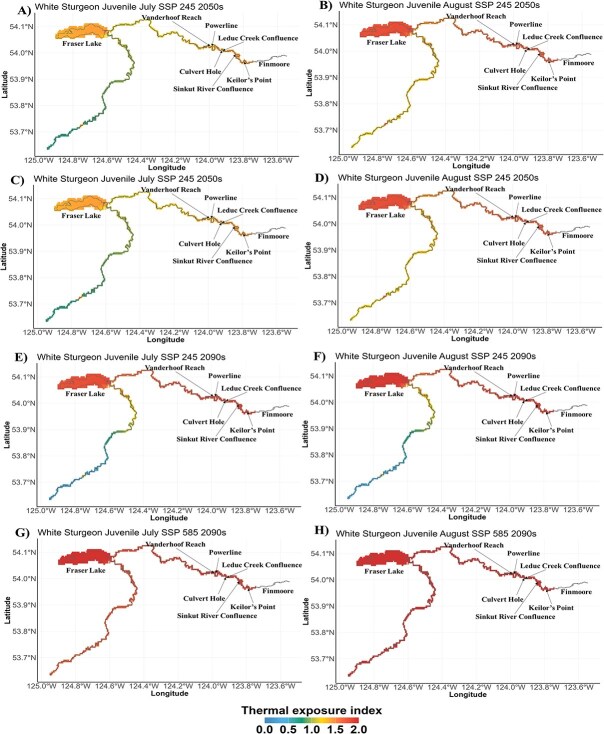
Thermal exposure risk spatial map for white sturgeon of the Nechako River life stages; juvenile under SSP2–4.5 and SSP5–8.5 by the 2050s (average 2050–59) and the 2090s (average 2090–99) for the STMP months (July and August). (A, B) Map for July and August under SSP2–4.5 by the 2050s. (C, D) Map for July and August under SSP5–8.5 by the 2050s. (E, F) Map for July and August under SSP2–4.5 by the 2090s. (G, H) Map for July and August under SSP5–8.5 by the 2090s. Cool and warm colours represent low and high thermal exposure risk, respectively.

By the 2090s, our analysis shows that the projected *T_e_* values under the SSP2-4.5, for embryo and yolk-sac larvae in the Vanderhoof Reach, were projected to remain <1 (Fig. 5C). In contrast, under SSP5-8.5, the *T_e_* values were projected to be slightly >1 for both life stages. Moreover, within the Vanderhoof Reach, *T_e_* was projected to be >1 for both feeding larvae scenarios under SSP2-4.5 and SSP5-8.5, respectively, although only slightly >1 under SSP2-4.5 scenario by the 2050s (Fig. 5C). As for the juvenile life stage, *T_e_* levels were projected to consistently exceed 1 in all critical habitats during July and August, irrespective of the SSP scenario (SSP2-4.5 and SSP5-8.5) (Fig. 5D, Fig. 7). Specifically, under SSP2-4.5, we projected *T_e_* values >1 for all critical habitats used by the juvenile life stage, except for Fraser Lake, in June. In July and August, all habitats were projected to have *T_e_* values well surpassing the optimal threshold, ranging from 1.78 in Fraser Lake to 1.91 in Powerline. Under SSP 5-8.5, habitats were projected to exhibit *T_e_* values exceeding 1 in June, July, August and September, with the lowest value of 1.50 in Fraser Lake (September) and the highest value of 2.0 observed in August across all habitats.

## Discussion

A key goal of water management is to balance societal needs with ecological diversity in freshwater ecosystems (Richter *et al.,* 2003; Geist, 2011; Mishra *et al.,* 2023). As climate change threatens these systems (Reid *et al.,* 2019; Rose *et al.,* 2023), understanding the interdependence between water management and climate impacts is important for informed decision-making and promoting ecological resilience. This study used an established framework (Oyinlola *et al.,* 2023) that combines a hydrological model and physiological data to evaluate the hydrothermal impact on different white sturgeon life stages under combined climatic and socio-economic scenarios. Results show that river temperatures in the Nechako are expected to rise significantly under SSP2-4.5 and SSP5-8.5 scenarios, especially in July, August and September, thereby increasing thermal exposure risks, particularly for the juvenile life stage.

The CEQUEAU hydrological–thermal model was selected for our study due to its effectiveness in predicting distributed water temperatures at the watershed scale (Khorsandi *et al.,* 2022) and its strong performance in simulating dam release operations and river thermal modelling (St-Hilaire *et al.,* 2015). The lower RMSE values at the Vanderhoof and Nautley stations indicate the model’s accuracy, although it tends to underestimate measured water temperatures. In contrast, the higher RMSE values at the Fort Fraser and Fort Fraser 13 s stations may be attributed to the complexities of river–lake transition such as thermal stratification and localized influences (Daigle *et al.,* 2009; Khorsandi *et al.,* 2022; Auffray *et al.,* 2023). Large nearby water bodies can also affect air temperature and humidity, further affecting model accuracy. These specific characteristics should be considered when applying the CEQUEAU model.

Our study found a consistent rise in the average water temperature of the Nechako River, expected to continue throughout the century under two climate change scenarios. Notable increases are projected for August (9 and 11% by the 2050s and 2090s) and September (16 and 33%) (Fig. 3). This warming trend aligns with findings in BC (Green *et al.,* 2014; Schnorbus *et al.,* 2014) and globally due to climate change (IPCC, 2023). Water temperature increases are expected to have adverse impacts on aquatic ecosystems and their biodiversity (Poff *et al.,* 2002; Palmer *et al.,* 2009; Cheung and Oyinlola, 2019). Our study highlights the importance of integrating species-specific details to enable modelling that provides time, life stage and location-specific evaluations.

Our study provides valuable insights into thermal stress faced by white sturgeon at different life stages and locations in the Nechako River. A *T_e_* value of 1 indicates an optimal temperature for white sturgeon, where their performance is at its peak (Fig. 1B). As *T_e_* values rise >1, there is an increased risk of thermal exposure, with higher *T_e_* values indicating more severe stress and reduced performance (Oyinlola *et al.,* 2023). In the 1980s, white sturgeon across all examined life stages experienced less thermal stress compared to projected conditions under climate change scenarios, as indicated by lower *T_e_* values except for juveniles where *T_e_* was projected to reach 1.14 in August. While the STMP aims to maintain river temperatures <20°C, the temperature management is restricted to periods determined by the presence of migrating sockeye salmon (Macdonald *et al.,* 2012). This could lead to water temperatures exceeding the 18°C threshold for the juveniles or warmer conditions outside the STMP’s active period, exposing other species to stress.

The analysis focused on specific locations rather than the whole river provided informative contrast. Habitat-specific evaluation in the 1980s, such as the Vanderhoof Reach, showed *T_e_* values <1 for the embryo, yolk-sac larvae and feeding larvae life stages. The Vanderhoof Reach has been identified as an important habitat for white sturgeon conservation in the Nechako River (SARA, 2002; Fisheries and Canada, 2014). However, except for Fraser Lake in July, all juvenile critical habitats showed *T_e_* values >1. Although remediating suitable substrates within key spawning habitats is essential to restoring recruitment (McAdam *et al.,* 2018), our results suggest that climate-related increases in water temperature could limit future recovery. Previous studies have noted the impact of thermal stress on the survival and growth of white sturgeon (Wang *et al.,* 1985; Boucher *et al.,* 2014; Earhart *et al.,* 2023). As such, the life stage species analysis in our study highlights stage-specific risks, which can guide both research and future mitigation.

Our study highlights that the embryonic and yolk-sac larvae life stages are less vulnerable to thermal risk than the feeding larvae (both feeding larvae18 and feeding larvae20) due to the differences in the time of year when these life stages are present. This finding is significant, as these earlier stages coincide with critical periods when substrate changes are associated with recruitment failure (McAdam, 2015; McAdam *et al.,* 2005). In the 2050s, the embryos and yolk-sac larvae are projected to experience a thermal risk of <1, regardless of climate change scenario. However, by the 2090s under the high-emission scenario (SSP5-8.5), the risk is projected to exceed 1 compared to the 1980s (Fig. 5). Such high thermal risk increases have significant impacts on the spawning, incubation and survival of white sturgeon (Wang *et al.,* 1985; Deng *et al.,* 2002), as increasing temperatures are linked to increased mortality rates and physical abnormalities (Van Eenennaam *et al.,* 2005; Leal *et al.,* 2021). These results show that the embryos and yolk-sac larvae display resilience, which provides valuable guidance for the timing of future mitigation. Feeding larvae will face high exposure across various climatic scenarios and time frames, especially during the warmer summer months when this life stage occurs. More studies are needed to understand how sturgeon larvae respond to changing conditions, especially given the lack of thermal tolerance studies for this life stage. Our model outcomes suggest that investigation of both peak temperatures and longer duration exposures would be informative.

Rising thermal risk for the juvenile stage emphasizes the need for improved thermal mitigation in regulated rivers (Cheng *et al.,* 2020). While the current STMP offers some protection during the summer for vulnerable migratory sockeye salmon, it may not adequately address the thermal impacts of climate change on multiple species. The ability for upstream water releases to achieve specific downstream mitigation targets (e.g. 20°C) is expected to diminish as rising temperatures increase reservoir outflow temperatures over the coming decades (Fig. 3). Such challenges are already becoming apparent because of the effects of drought in 2023 and 2024 (CBC News, 2024), as limitations on reservoir refilling under sequential years of drought will limit the ability to provide summer cooling flows if drought conditions continue. While other recovery measures aim to mitigate the causes of recruitment collapse, our findings indicated that climate warming poses significant challenges for future species recovery. Further research is needed to refine thermal tolerance for various life stages and understand the timeline of these risks.

### Study limitations

Our methodology integrates a spatially distributed hydrological model with the physiological tolerance limits of white sturgeon’s early life stages under climate change scenarios. This framework allows us to assess climate-induced impacts on white sturgeon in the Nechako River, enabling a detailed examination of the thermal vulnerability across specific river reaches relevant to different life stages. Nonetheless, it is important to acknowledge the inherent limitations of our approach.

Our water temperature prediction model may face accuracy issues due to uncertainties in the input meteorological and observed water temperature data used for calibration (Khorsandi *et al.,* 2022; Yoshida *et al.,* 2022). The model’s precision is influenced by the resolution of physical catchment properties and the aggregation or disaggregation process (Markhali *et al.,* 2022). While there is no significant systematic bias in our simulations, the CEQUEAU model tends to underestimate the extreme temperatures, which could downplay impacts. The biases are linked to some potentially faulty calibration data and the presence of large water bodies (Khorsandi *et al.,* 2023). As ecosystem health deteriorates and weather systems become more unstable, the predictive accuracy of current models may decline. This highlights the importance of regular validation and updates to account for emerging patterns. Careful consideration of the data preparation step is essential to address these challenges and improve the accuracy of the model.

Predicting the optimal temperature and *T_e_* for white sturgeon in the Nechako River based on laboratory data has limitations due to the complexity of ecosystems and species–environment interactions. For instance, the optimal temperature range of 14 and 18°C (Table 1) contrasts with the broader natural spawning range of 11–18°C (Sykes and Bio, 2010). Additionally, juvenile survival remains high even when summer temperatures exceed a *T_e_* threshold of 18°C (Buckner *et al.,* 2024). These discrepancies highlight the necessity for a more ecologically inclusive approach to evaluate thermal limits and exposure risks for white sturgeon. Relying on data from short-term laboratory studies data does highlight areas of concern but may also overlook factors that influence responses in the natural environment, including acclimation and adaptation to elevated temperatures, potentially leading to misinformed predictions and management decisions. Natural thermal variations should be considered when evaluating the physiological performance of wild species (Morash *et al.,* 2018).

Our framework’s inability to account for the frequency and duration of juvenile and adult movements, such as seeking cooler areas or deeper waters, limits its effectiveness. This may underestimate the species’ resilience and adaptive strategies, reducing its applicability for real-time conservation and management decisions.

## Conclusion

Our study highlights the significant impact of climate change and water management practises on the optimal thermal performance and survival of white sturgeon in the Nechako River, British Columbia. Using the CEQUEAU hydrological–thermal model, we projected future water temperatures and assessed the *T_e_* for early life stages of white sturgeon under two climate scenarios (SSP2-4.5 and SSP5-8.5). A *T_e_* value of 1 signifies the assumed optimal temperature for white sturgeon, where their performance is at its peak. When *T_e_* values exceed 1, it denotes a higher risk of thermal exposure, with higher *T_e_* values suggesting more severe stress and reduced performance. Embryos and yolk-sac larvae showed resilience to water temperature increases whereas feeding larvae and juveniles experienced significantly higher *T_e_* values, often exceeding 1. Modelling outcomes show that current water management under the STMP will not adequately address the thermal requirements of white sturgeon under projected future climate conditions. Increasing *T_e_* values for feeding larvae and juveniles highlight the need to include white sturgeon in future thermal mitigation strategies. Overall, our study provides valuable insights into the complex relationship between water management, climate change and the thermal ecology of white sturgeon in the Nechako River, emphasizing the need for proactive and adaptive conservation measures.

## Supplementary Material

Web_Material_coaf014

## Data Availability

The data that supports the findings of this study are openly available at https://doi.org/10.5683/SP3/EK7Y7Y.
