# Supplementary material for: Assessing the impact of climate change and a water management programme on white sturgeon physiology in the Nechako River, British Columbia
Source: Conserv Physiol. 2025 Mar 8;13(1):coaf014. doi: 10.1093/conphys/coaf014 (PMC11891139; doi:10.1093/conphys/coaf014)
Supplement: Web_Material_coaf014 [file web_material_coaf014.pdf]

Title: Assessing the impact of climate change and a water management program on white sturgeon physiology in Nechako River, British Columbia

Supplementary Information

CEQUEAU model: Modelling Nechako River water temperature

CEQUEAU is a hydrological and water temperature model that is semi-distributed (Khorsandi et al., 2022; Morin & Couillard, 1990; St-Hilaire et al., 2015) (Fig 1B). The hydrological module of CEQUEAU includes snowmelt and evapotranspiration formulations as well as conceptual water storage in two soil horizons, while the thermal module calculates the surface heat budget within each model grid cell. Land cover and topography are required as input physiographic data. The 10-meter resolution land cover data provided by ESRI, and the European Space Agency (ESA) (Karra et al., 2021; Zanaga et al., 2022) is used in this study, the NASA SRTM Digital Elevation Model (DEM) with a 30-meter resolution (Farr et al., 2007) is used for topography. Aside from physiographic data, meteorological data are also needed for the model, including daily solid and liquid precipitation and maximum and minimum daily air temperature.

To compute surface heat fluxes (incoming shortwave radiation, net longwave radiation, latent heat, and sensible heat), additional meteorological input variables such as solar radiation, wind velocity, vapour pressure, and cloud cover are used to calculate the heat budget. At each time step, the model also accounts for heat adverted from upstream, local runoff and interflow, as well as groundwater.

The physical characteristics of the watershed are accounted for by the model through decomposition into Elementary Representative Areas (ERA) of equal surface, referred to

as “whole squares”. The ERA characteristics include altitude, forest cover percentage, and the percentage of lakes and wetlands coverage. To define water routing, the ERAs are further subdivided into a maximum of four “partial squares”, based on altitude, slopes, and water divides. At each time step (daily), a hydrological budget is calculated for each partial square, and water availability for runoff is apportioned proportionally to the partial square areas while identifying the downstream receiving partial square. In CEQUEAU, the hydrological module output is utilised as input for the thermal module. The thermal module computes various heat fluxes, such as direct shortwave radiation, latent heat, longwave radiation, convection, advection from upstream, local runoff, interflow, and groundwater inflows. The surface heat budget is calculated using Equation 1.:

$$\Delta H = H_{short} + H_{long} + H_{ET} + H_{sens} + H_{ups} + H_{downs} + H_{local} + H_{ground} \quad (1)$$

WhereΔH is the change in overall heat for a CP (MJ); H_{short} is incoming shortwave radiation; H_{long} is heat gain/loss by longwave radiation; H_{ET} is the latent heat loss (evaporation); H_{sens} is the sensible heat gain/loss by convection; H_{ups} and H_{downs} are respectively the heat gain from upstream and heat loss to the downstream; H_{local} is the heat from local runoff and interflow; and H_{ground} is the heat term related to groundwater. Finally, the thermal module calculates water temperature change using the following equation:

$$\Delta T = \frac{\Delta H}{V.\theta} \quad (2)$$

where ΔT is the change in the water temperature (°C); V is the estimated water volume by the hydrological module (m^3), and θ is the heat capacity of water (4.187$\frac{MJ}{m^3.°C}$) (Guy Morin & Paquet, 2007)..

CEQUEAU model: modelling historical temperature and future climate change scenarios.

To begin implementing CEQUEAU, the model's parameters must first be calibrated. The hydrological module is calibrated using observed streamflow data from hydrometric stations along the Nechako River. The thermal module is then calibrated using water temperature gauges located between the dam and Vanderhoof. The parameters are manually calibrated before applying an automatic calibration algorithm called the Covariance Matrix Adaptation Evolution Strategy (CMA-ES) (Hansen, 2006). Additionally, the multi-site temperature calibration method of Khorsandi et al. (2022) is used to adjust the parameters of the water temperature module.

To project future changes in the Nechako River water temperature and water flow, and to account for variability in CEQUEAU model output, data from eight General Circulation Models (GCMs), part of the Coupled Model Intercomparison Project Phase 6 (CMIP6) (Eyring et al., 2016), were used (Table 2). This ensemble approach is to quantify and capture uncertainty that may arise due to each model's assumptions and approximations. For this study, we examined two Shared Socio-economic Pathways (SSP) scenarios. These scenarios include SSP2-4.5 (the intermediate scenario), which describes a “middle of the road” scenario where the world follows historical, social, and economic trends (O’Neill et al., 2017); also, the radiative forcing level stabilises at 4.5 W/m^2 before 2100. The second scenario is SSP5-8.5 (the high emission scenario), which describes a world with rapid economic growth and faith in competitive markets, innovation, and participatory societies (O'Neill et al., 2017), and the radiative forcing level increased at 8.5 W/m^2 by 2100 with no mitigation.

Each global climate model utilised the SSP forcings to provide meteorological variables required for the CEQUEAU model execution. These datasets underwent bias correction by applying the N-dimensional Multivariate Bias Correction algorithm (MBCn) (Cannon, 2018) on the reference period of 1981-2010, employing ERA5 data as the baseline dataset. The bias correction was then applied to the climate model data for each of the 8 climate models, two radiative forcing scenarios (4.5 and 8.5 W/m^2), and horizon. The bias correction protocol was implemented at 3-hour intervals, providing a more accurate representation of the meteorological diurnal cycle and enhancing the precision of daily averages (Gatien et al., 2022). However, this also limited the number of available GCMs as not all GCMs in CMIP6 archive data at the sub-daily time step.

Table S1: List of Earth System Models that are part of the Coupled Model Intercomparison Project Phase 6 (CMIP6) used in this study.

**Model**	**Full name**	**Spatial resolution**	**Reference**
BCC-CSM2-MR	Beijing Climate Centre Climate System Model	110 x 110km	(Wu et al., 2020)
CMCC-CM2-SR5	Euro-Mediterranean Centre on Climate Change coupled climate model	0.9° Lat x 1.25° Lon	(Lovato et al., 2022; Lovato & Peano, 2020)
CMCC-ESM2	Second-generation CMCC Earth System Model	0.9° Lat x 1.25° Lon	(Lovato et al., 2022; Lovato &

			Peano, 2020)
EC-Earth3	European Centre Earth3 Model	40 x 40 km	(Döscher et al., 2021)
MIROC6	Model for Interdisciplinary Research on Climate	1.4° Lat x 1.4° Lon	(Kawamiya et al., 2020)
MPI-ESM1-2-HR	Max Planck Institute for Meteorology Earth System Higher-resolution Model	100 x 100 km	(Gutjahr et al., 2019; Müller et al., 2018)
MPI-ESM1-2-LR	Max Planck Institute for Meteorology Earth System Lower-resolution Model	200 x 200 km	(Wieners et al., 2019)
MRI-ESM2-0	Meteorological Research Institute Earth System Model version 2.0	100 x 100 km	(Yukimoto et al., 2019)

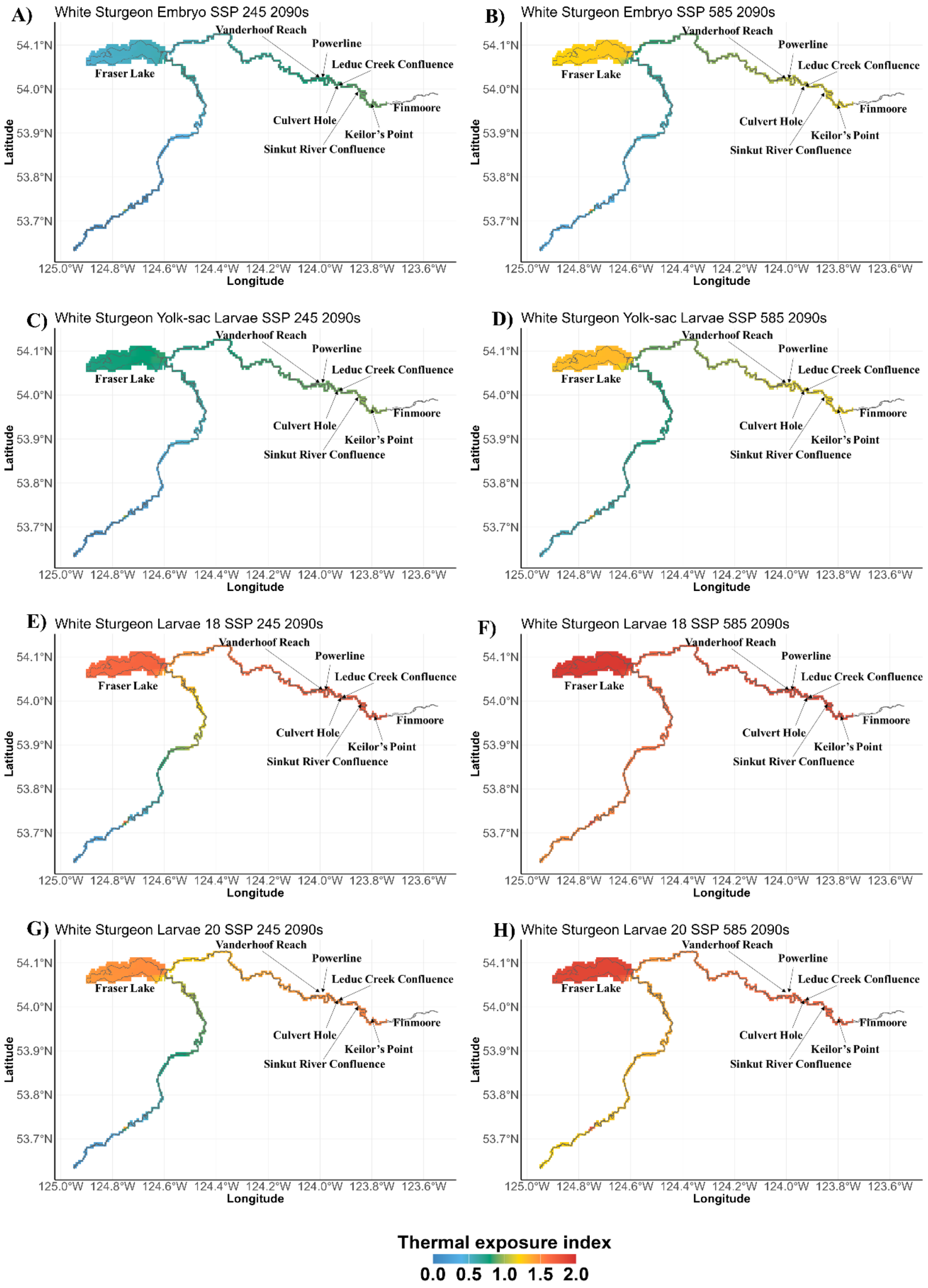

Fig S1: Thermal exposure risk spatial map for Nechako River white sturgeon life stages; embryo, yolk-sac larvae and larvae under SSP2-4.5 and SSP5-8.5 in the 2090s (average

2090-2009). (A, B) For the embryo life stage occurring between May 15th to June 24th. (C, D) For the yolk-sac larvae life stage occurring between May 24th to July 7th. (E, F, G, H) For the larvae life stage occurred between June 20th to July 31st. Cool and warm colours represent low and high thermal exposure risk, respectively.

Reference

Cannon, A. J. (2018). Multivariate quantile mapping bias correction: An N-dimensional probability density function transform for climate model simulations of multiple variables. *Climate Dynamics*, *50*, 31–49.

Eyring, V., Bony, S., Meehl, G. A., Senior, C. A., Stevens, B., Stouffer, R. J., & Taylor, K. E. (2016). Overview of the Coupled Model Intercomparison Project Phase 6 (CMIP6) experimental design and organization. *Geoscientific Model Development*, *9*(5), 1937–1958.

Farr, T. G., Rosen, P. A., Caro, E., Crippen, R., Duren, R., Hensley, S., Kobrick, M., Paller, M., Rodriguez, E., & Roth, L. (2007). The shuttle radar topography mission. *Reviews of Geophysics*, *45*(2).

Gatien, P., Arsenault, R., Martel, J.-L., & St-Hilaire, A. (2022). Using the ERA5 and ERA5-Land reanalysis datasets for river water temperature modelling in a data-scarce region. *Canadian Water Resources Journal/Revue Canadienne Des Ressources Hydriques*, 1–18.

Hansen, N. (2006). The CMA evolution strategy: A comparing review. *Towards a New Evolutionary Computation: Advances in the Estimation of Distribution Algorithms*, 75–102.

Karra, K., Kontgis, C., Statman-Weil, Z., Mazzariello, J. C., Mathis, M., & Brumby, S. P. (2021). *Global land use/land cover with Sentinel 2 and deep learning*. 4704–4707.

Khorsandi, M., St-Hilaire, A., & Arsenault, R. (2022). Multisite calibration of a semi-distributed hydrologic and thermal model in a large Canadian watershed. *Hydrological Sciences Journal*, *67*(14), 2147–2174.

Morin, G., & Couillard, D. (1990). Predicting river temperatures with a hydrological model. *Encyclopedia of Fluid Mechanic, Surface and Groundwater Flow Phenomena*, *10*, 171–209.

O'Neill, B. C., Kriegler, E., Ebi, K. L., Kemp-Benedict, E., Riahi, K., Rothman, D. S., Van Ruijven, B. J., Van Vuuren, D. P., Birkmann, J., & Kok, K. (2017). The roads ahead: Narratives for shared socioeconomic pathways describing world futures in the 21st century. *Global Environmental Change*, *42*, 169–180.

St-Hilaire, A., Boucher, M.-A., Chebana, F., Ouellet-Proulx, S., Zhou, Q. X., Larabi, S., Dugdale, S., & Latraverse, M. (2015). *Breathing a new life to an older model: The CEQUEAU tool for flow and water temperature simulations and forecasting*. Proceedings of the 22nd Canadian Hydrotechnical Conference.

Zanaga, D., Van De Kerchove, R., Daems, D., De Keersmaecker, W., Brockmann, C., Kirches, G., Wevers, J., Cartus, O., Santoro, M., & Fritz, S. (2022). *ESA WorldCover 10 m 2021 v200*.
